# Prediction Models of Severity in Acute Biliary Pancreatitis

**DOI:** 10.3390/diagnostics15020126

**Published:** 2025-01-07

**Authors:** Iulia Ratiu, Renata Bende, Camelia Nica, Oana Budii, Calin Burciu, Andreea Barbulescu, Tudor Moga, Bogdan Miutescu, Roxana Sirli, Mirela Danila, Alina Popescu, Felix Bende

**Affiliations:** 1 Department of Gastroenterology and Hepatology, “Victor Babes” University of Medicine and Pharmacy Timisoara, 300041 Timisoara, Romania; ratiu.iulia@umft.ro (I.R.); camelia.foncea@umft.ro (C.N.); oana.budii@yahoo.com (O.B.); moga.tudor@umft.ro (T.M.); miutescu.bogdan@umft.ro (B.M.); sirli.roxana@umft.ro (R.S.); danila.mirela@umft.ro (M.D.); popescu.alina@umft.ro (A.P.); bende.felix@umft.ro (F.B.); 2 Advanced Regional Research Center in Gastroenterology and Hepatology, “Victor Babes” University of Medicine and Pharmacy Timisoara, 300041 Timisoara, Romania; calin.burciu@umft.ro (C.B.); barbulescu.andra91@gmail.com (A.B.); 3 Department of Gastroenterology, Faculty of Medicine, Pharmacy and Dental Medicine, “Vasile Goldis” West University of Arad, 310414 Arad, Romania

**Keywords:** acute biliary pancreatitis, severity prediction, multiparametric models, comparison

## Abstract

**Background:** Acute pancreatitis is a common condition with a variable prognosis. While the overall mortality rate of acute pancreatitis is relatively low, ranging between 3 and 5% in most cases, severe forms can result in significantly higher morbidity and mortality. Therefore, early risk assessment is crucial for optimizing management and treatment. The aim of the present study wasto compare simple prognostic markers and identify the best predictors of severity in patients with acute pancreatitis. **Material and Methods:** A retrospective analysis was carried outon 108 patients admitted in our center during one year with acute biliary pancreatitis. Acute pancreatitis severity was stratified based on the revised Atlanta criteria. **Results:** 108 subjects (mean age of 60.1 ± 18.6, 65.7% females) diagnosed with acute biliary pancreatitis were included. Based on the Atlanta criteria, 59.3% (64/108) of the subjects were classified as having mild acute biliary pancreatitis, 35.2% (38/108) as having a moderate–severe pancreatitis, and 5.5% (6/108) were classified as having severe acute pancreatitis. In univariate analysis, the following parameterswere associatedwith at least a moderate–severe form of acute pancreatitis: Balthazar score, fasting blood glucose (mg/dL), modified CTSI score, CRP values at 48 h, BISAP score at admission, CTSI score, Ranson score, duration of hospitalization (days), and the presence of leukocytosis (×1000/µL) (all *p* < 0.05).BISAP score at admission (AUC-0.91), CRP levels at 48 h (AUC-0.92), mCTSI (AUC-0.94), and CTSI score (AUC-0.93) had the highest area under the curve (AUC) for predicting the severity of acute pancreatitis. In multivariate analysis, the model including the following independent parameters was predictive for the severity of acute pancreatitis: CTSI score (*p* < 0.0001), BISAP score (*p* = 0.0082), and CRP levels at 48 h (*p* = 0.0091), respectively. The model showed a slightly higher AUC compared to the independent predictors (AUC-0.96). **Conclusions:** The use of a multiparametric prediction model can increase the accuracy of predicting severity in patients with acute biliary pancreatitis.

## 1. Introduction

Acute pancreatitis (AP) is an inflammatory disease of the pancreas with a variable clinical course, ranging from mild to severe cases involving multiple organ failure and potential mortality. Among the various causes, acute biliary pancreatitis (ABP) is particularly common, and it is often triggered by small gallstones or biliary sludge obstructing the ampulla of Vater [1,2]. Pancreatic hyperstimulation and ductal obstruction play a central role in the pathophysiology of the disease, as they elevate ductal pressure and drive unregulated trypsin activation within pancreatic acinar cells. This cascade disrupts normal protective mechanisms, leading to severe local inflammation and, in critical cases, systemic complications. Some other factors can contribute to the pathogenesis of ABP, such as anatomical variations, genetic predisposition, and abnormal bile reflux, underlining its complex and multifactorial nature [2].

Diagnostic strategies for biliary etiology in AP include both laboratory markers and imaging techniques. Already known and validated serum markers such as CPR at 48 h, creatinine at 48 h, BUN, and neutrophil-to-lymphocyte ratio have proven to be accurate in predicting the severity of acute pancreatitis regardless of etiology. Elevated serum alanine aminotransferase (ALT > 1.0 µkat/L) strongly predicts gallstone pancreatitis with a high positive predictive value [2,3,4]. The Ranson criteria and the Bedside Index for Severity in Acute Pancreatitis (BISAP) score are among the most commonly utilized tools in routine clinical practice [5,6]. Imaging modalities, such as endoscopic ultrasonography, are essential for identifying gallstones and confirming biliary obstruction. At the same time, transabdominal ultrasound and magnetic resonance cholangiopancreatography (MRCP) provide non-invasive visualization of biliary stones or sludge within the bile duct. Computer tomography can confirm the diagnosis and identify the cause, but most importantly, it can provide valuable information about complications and prognosis [3,4].

Given the unpredictable clinical trajectory of AP, early and accurate assessment of severity is essential for guiding treatment and improving patient outcomes. Prediction models and scoring systems, such as BISAP, Ranson, and CTSI, are crucial for stratifying patients by risk and tailoring management accordingly. These tools help clinicians identify high-risk ABP cases promptly, facilitating timely interventions and potentially reducing complications. As the incidence of AP continues to rise, with rates reported between 13 and 45 cases per 100,000 people in regions like the USA, Western Europe, and Japan, the development and refinement of reliable prediction models are increasingly important [4,7].

The study aims to identify which of the validated predictors for predicting the severity of acute pancreatitis has the best prediction accuracy and if the combined use of several predictors can increase the accuracy. Enhanced understanding of severity predictors and more targeted use of risk scores may lead to better therapeutic strategies which hold promise for improving outcomes in biliary pancreatitis.

## 2. Materials and Methods

### 2.1. Subjects Selection

We conducted a retrospective study that included patients diagnosed with acute biliary pancreatitis over a one-year period. In our clinic, 188 cases of acute pancreatitis were admitted, of which 57.7% (108) were of biliary etiology, 28.7% (54) were of alcoholic etiology, 8.5% (16) were due to hypertriglyceridemia, 2.7% (5) were post-ERCP pancreatitis, 1 case was of autoimmune etiology, and in 2.1% (4) of cases, a definitive etiology could not be established.

Acute pancreatitis (AP) was diagnosed when at least two of the following criteria were fulfilled: characteristic abdominal pain with typical localization, serum lipase levels exceeding three times the upper normal limit, and imaging findings indicative of AP.

Patients were included based on a comprehensive clinical evaluation and when they met the specific inclusion criteria for ABP, as confirmed by clinical, laboratory, and imaging findings. Patients with acute pancreatitis of non-biliary etiology (e.g., alcohol-induced, hypertriglyceridemia, or idiopathic cases) were excluded from the study. Exclusion criteria included patients with chronic pancreatitis (evidenced by intraductal calculi, ductal strictures, or parenchymal calcifications), those presenting with infections such as cholangitis or cholecystitis, pregnant women, and individuals under the age of 18.

Patients were classified by disease severity according to the revised Atlanta criteria, which classify acute pancreatitis into three categories: mild acute Pancreatitis (MAP), characterized by the absence of organ failure and complications; moderately severe acute pancreatitis (MSAP), defined by transient organ failure that resolves within 48 h or the presence of local complications; and severe acute pancreatitis (SAP), marked by persistent organ failure lasting longer than 48 h [8].

### 2.2. Data Collection

Upon admission, each patient underwent a comprehensive clinical assessment, including anthropometric measurements and standard laboratory tests. These tests included serum lipase and C-reactive protein (CRP) to assess inflammatory status and complete blood count (CBC). Liver function tests were conducted to measure total bilirubin (TB), alkaline phosphatase (ALP), gamma-glutamyl transferase (GGT), aspartate aminotransferase (AST), and alanine aminotransferase (ALT). Additional tests included serum creatinine, fibrinogen levels, coagulation profile, serum electrolytes, triglycerides, and fasting blood glucose. Imaging studies were conducted to confirm biliary involvement; all patients underwent a B-mode abdominal ultrasound using a LOGIQ E10 ultrasound machine (GE Healthcare, Wauwatosa, WI, USA), to evaluate gallstones and biliary ductal status, and abdominal computed tomography (CT) scans were conducted to evaluate pancreatic morphology and detect potential complications.

### 2.3. Scoring System

The following prognostic scores were employed to evaluate disease severity and predict outcomes in acute biliary pancreatitis (ABP):

#### 2.3.1. Balthazar Score/CTSI Score

Balthazar score assesses pancreatic inflammation and necrosis based on CT imaging. It assigns points based on CT findings, grading the pancreas from normal (Grade A) to severe (Grade E). Points are added according to the percentage of necrosis and peri-pancreatic fluid collections. Higher grades are correlated with increased severity. To determine the statistical analysis, the Balthazar score was categorized numerically, assigning a grade from 1 to 5 depending on the severity, 1 corresponding to grade A and 5 to grade E [7].

#### 2.3.2. Modified Computed Tomography Severity Index (mCTSI)

The mCTSI is an extension of the original CT Severity Index or Balthazar score, combining pancreatic inflammation and necrosis with extrapancreatic complications. The score ranges from 0 to 10. Points are assigned based on pancreatic abnormalities, the extent of necrosis, and the presence of complications such as fluid collections or ascites [7].

#### 2.3.3. BISAP Score (Bedside Index for Severity in Acute Pancreatitis)

The BISAP score is a five-point system used at the bedside to predict severity within 24 h of admission by assigning one point for the following parameters: age > 60 years, pleural effusion, BUN > 25 mg/dL, impaired mental status, and presence of systemic inflammatory response syndrome (SIRS). A higher BISAP score correlates with an increased risk of severe pancreatitis and mortality [7].

#### 2.3.4. Ranson Score

The Ranson score estimates mortality of patients with pancreatitis based on initial and 48 h lab values. This score assesses severity using 11 clinical and laboratory criteria. At admission, five criteria are assessed: white blood cell count > 16,000/mm^3^, blood glucose > 200 mg/dL, serum lactate dehydrogenase (LDH) > 350 IU/L, age > 55 years, and AST > 250 IU/L. Six additional criteria are evaluated within 48 h: hematocrit decrease > 10%, arterial PO_2_ < 60 mm Hg, base deficit > 4 mEq/L, BUN increase > 5 mg/dL, serum calcium < 8 mg/dL, and fluid sequestration > 6 L. Each criterion counts as one point; higher scores indicate greater severity and higher mortality risk [7].

Each score was assessed for its predictive value regarding the progression to moderate or severe forms of AP. To enhance the assessment of disease severity, additional parameters such as CRP levels at 48 h, fasting blood glucose, and white blood cell count were also contained in the analysis.

### 2.4. Statistical Analysis

Data analysis was carried outusing the MedCalcprogram, Version 19.4 (MedCalc Software Corporation, Brunswick, ME, USA) and Microsoft Office package, Excel 2019 (Microsoft for Windows). Descriptive statistics were used to summarize the clinical data of the patients. The Kolmogorov–Smirnov test was applied to assess the distribution of numerical variables. Continuous variables with a normal distribution were expressed as mean ± standard deviation (SD), while those with a non-normal distribution were reported as median and interquartile range (IQR). Categorical variables were presented as frequencies and percentages.

The groups were analyzed using the Kruskal–Wallis H test, followed by posthoc comparisons performed with the Mann–Whitney U test and adjusted using the Bonferroni correction. For continuous variables with normal distribution, Student’s *t*-test was used, while the Mann–Whitney *U* test was applied for those with non-normal distribution.

Spearman’s rank-order correlation was employed to explore the relationship between laboratory parameters or prognostic scores and the severity of ABP, alongside Pearson’s χ2-test for categorical variable analysis. Univariate regression and multivariate logistic regression analyses were operated to identify independent predictors of ABP severity and to test the predictive accuracy of the mentioned predictors for identifying cases of at least moderate–severe acute pancreatitis.

The Akaike information criterion (AIC) was utilized to select the optimal regression model. A *p*-value < 0.05 was considered statistically significant.

## 3. Results

Our patient cohort included 108 subjects, aged between 19 and 93 years, with a mean age of 60.1 ± 18.6 years, diagnosed with acute biliary pancreatitis. Females represented 65.7% (71/108) of the cohort. At admission, 69.4% (75) of the subjects had ultrasound-proven gallstones, 9.3% (10) had biliary sludge, 13.9% (15) had previously undergone cholecystectomies, and 7.4% (8) were without ultrasound-detected gallstones. A total of 28.7% (31) of the subjects also had associated obstructive jaundice or anicteric biliary obstruction, having choledochal lithiasis, and underwent ERCP within the first 24 h of admission. Regarding cholecystectomy, 13.9% (15 patients) had undergone cholecystectomy prior to the study period, while the remaining 86.1% (93 patients) underwent cholecystectomy after the episode of acute pancreatitis. Of these, 81 out of 93 (87.1%) underwent cholecystectomy during the same hospitalization after being transferred to the Surgery Clinic following symptom resolution. The rest of the 12.9% (12) underwent cholecystectomy at a later time, as they either had severe forms of pancreatitis or significant collections. The demographic characteristics and laboratory results are summarized in Table 1.

**Figure 1:**
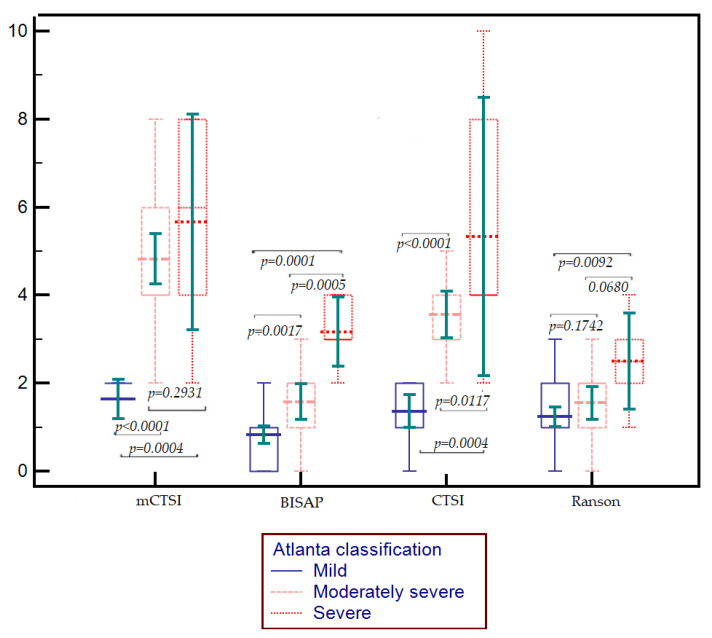
Boxplot illustrating the median differences in mCTSI, BISAP, CTSI, and Ranson score according to the severity of the biliary acute pancreatitis.

Based on the Atlanta criteria, 59.3% (64/108) of the subjects were classified as having mild acute biliary pancreatitis, 35.2% (38/108) as having a moderate–severe pancreatitis, and 5.5% (6/108) were classified as having severe acute pancreatitis (Figure 1).

By analyzing the potential associations between the Atlanta classification of the subjects with biliary acute pancreatitis and some laboratory parameters, as well as between Atlanta classification and various previously validated scores for predicting the severity of biliary acute pancreatitis, we evidenced statistically significant correlations between Atlanta classification of acute pancreatitis and mean BMI (kg/m^2^) values, AST values, ALT values, TB levels, fasting blood glucose, CRP values, white blood cells, CRP-48, serum creatinine, serum creatinine at 48 h, serum urea, BUN, procalcitonin levels, BISAP, CTSI score, mCTSI score, Ranson score, and the duration of hospitalization (days). The power of the association between the previously mentioned parameters is summarized in Table 2.

We determined the median values of the following scores: Ranson, BISAP, mCTSI, CTSI. It was observed that there is a statistically significant difference between the median values in patients with mild forms of acute biliary pancreatitis compared to those with moderate–severe or severe forms (all *p* < 0.05). This was observed for all scores with the exception of the Ranson score, where no statistically significant difference was evident between the mild form compared to the moderate–severe one (*p* = 0.1742) (Figure 1). Regarding the differentiation of moderate–severe and severe forms, only the BISAP and CTSI scores showed significantly higher values in patients with severe pancreatitis compared to those with moderate–severe (*p* = 0.0005 and *p* = 0.01174, respectively), while for the Ranson score and mCTSI, there were no significant differences between their values in patients with moderate–severe forms compared to those with severe forms (0.0680 and 0.2931, respectively) (Figure 1).

Univariate analysis revealed that the following parameterswere associatedwith at least a moderate–severe form of acute pancreatitis: fasting blood glucose (mg/dL) (*p* = 0.0001), modified CTSI score (*p* < 0.0001), CRP values at 48 h (*p* = 0.0004), BISAP score at admission (*p* < 0.0001), CTSI score (*p* = 0.001), Ranson score (*p* = 0.002), and duration of hospitalization (days) (*p* = 0.006).

**Figure 2:**
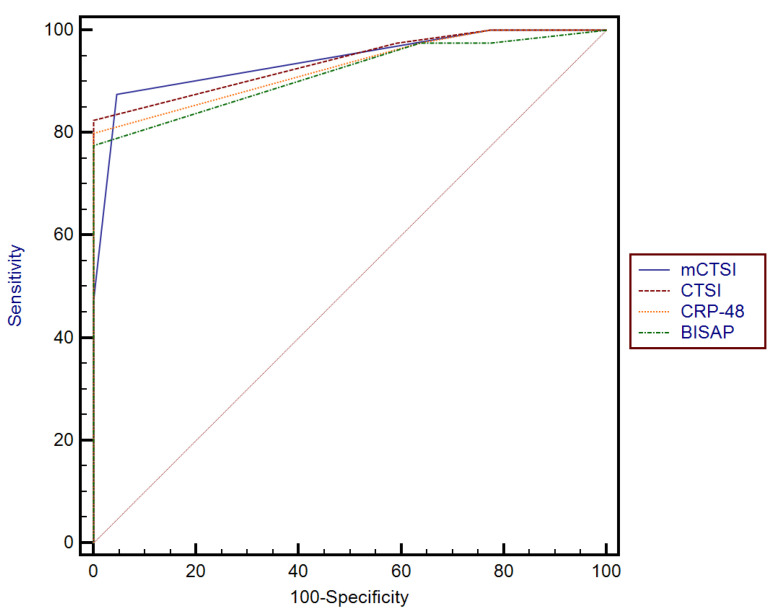
Comparison between receiver operating characteristic curve for mCTSI, BISAP, CTSI, and CRP-48 scores.

The accuracy of predicting the severity of acute pancreatitis of these parameters was analyzed, and the BISAP score at admission (AUC-0.91), CRP levels at 48 h (AUC-0.92), mCTSI (AUC-0.94), and CTSI score (AUC-0.93) had the highest area under the curve (AUC) for predicting the severity of acute pancreatitis (Figure 2).

In multivariate analysis, the model including the following independent predictors was associated with the severity of acute pancreatitis: CTSI score (*p* < 0.0001), BISAP score (*p* = 0.0082), and CRP levels at 48 h (*p* = 0.0091), respectively (Table 3).

**Figure 3:**
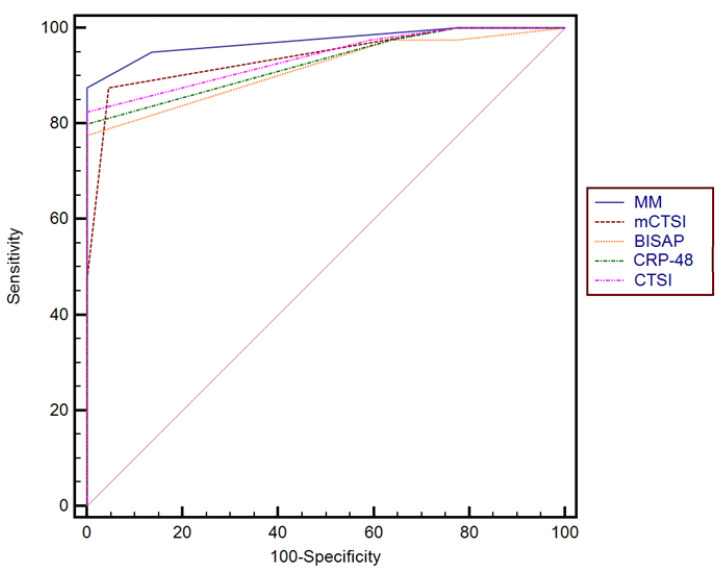
Comparison between receiver operating characteristic curve for the multiparametric model (MM), mCTSI, BISAP, CTSI, and CRP-48 scores.

Using these factors as predictors, by multiple regression analysis we obtained a multiparametric model (MM) for predicting the severity of biliary acute pancreatitis. The model showed a slightly higher AUC compared to the independent predictors (AUC-0.96) (see Figure 3).

Although the difference between the prediction accuracy of the independent scores compared to the multiparametric model (MM) was not significant (Table 4), when we performed the classification of 40 randomly chosen and blinded subjects from our group, the percentage of patients correctly classified using the multiparametric model was higher compared to using CTSI (*p* = 0.028), BISAP (*p* = 0.028), or CRP-48 (*p* = 0.015)scores independently (Table 5). Furthermore, the multiparametric model demonstrated superior diagnostic performance, achieving the highest sensitivity (97.5%) and negative predictive value (NPV, 97%) compared to individual markers: BISAP (sensitivity of 87.3%, NPV of 84%), CTSI (sensitivity of 81.1%, NPV of 78.2%), mCTSI (sensitivity of 80%, NPV of 77.4%), and CRP at 48 h (sensitivity of 86%, NPV of 81.3%).

## 4. Discussion

The present study aimed to assess the ability of various laboratory markers and clinical scores to evaluate the severity of acute biliary pancreatitis (ABP). Our findings show that multiple parameters, including BMI, AST, ALT, total bilirubin, fasting blood glucose, CRP, white blood cell count, serum creatinine, procalcitonin levels, and clinical scoring systems such as BISAP, CTSI, mCTSI, and Ranson, are significantly associated with ABP severity as defined by the Atlanta classification.

A notable finding of this study was the differential effectiveness of various scoring systems in distinguishing between mild, moderate–severe, and severe ABP cases. While BISAP and CTSI score values were effective in differentiating severe cases from moderate–severe cases, the Ranson and mCTSI scores were less precise for this purpose.

Ranson score did not show significant differences between the mild and moderate–severe pancreatitis groups (*p* = 0.1742); similarly, there were no significant differences between moderate–severe and severe groups (*p* = 0.0680). While being very often used to assess the severity of acute pancreatitis, the Ranson score may not effectively differentiate between certain severity levels in ABP, particularly in the context of early disease stages or between moderate–severe and severe cases. In a study involving 161 patients, Cho et al. [7] demonstrated that the Ranson score showed excellent sensitivity (85.7%) and negative predictive value (95.3%). However, its specificity and positive predictive value (PPV) were relatively low (44.4% and 18.8%, respectively). These findings indicate an overestimation of severe acute pancreatitis (AP) when using the Ranson score, with approximately 80% of patients scoring above 3 not having severe AP.

On the other hand, other studies support the ongoing utility of the Ranson score in stratifying severity and predicting mortality in AP, particularly when used in conjunction with other tools [9].

In our study, the accuracy of predicting the severity of acute pancreatitis was analyzed, and BISAP score at admission (AUC-0.91), CRP levels at 48 h (AUC-0.92), mCTSI (AUC-0.94), and CTSI score (AUC-0.93) had the highest area under the curve (AUC) for predicting the severity of acute pancreatitis.

Our findings are also supported by other studies.In a prospective study of 50 patients with acute pancreatitis, the BISAP score demonstrated an accuracy of 84% in predicting SAP, exceeding the 76% accuracy of serum procalcitonin (when a cut-off value of ≥3.29 ng/mL was used) and matching the performance of the APACHE II score. Furthermore, logistic regression showed that BISAP had the best predictive significance [10].

In a meta-analysis involving 1972 subjects, Yang and Li assessed the diagnostic accuracy of BISAP in predicting SAP. They concluded that while BISAP demonstrated high specificity, its low sensitivity limited its utility as a standalone tool for evaluating AP severity [11]. A multicenter validation study is needed to confirm these findings and further clarify BISAP’s role in assessing AP.

A meta-analysis of 30 studies [12], encompassing data from 5988 cases of acute pancreatitis, reported pooled area under the curve (AUC) values for mortality prediction, as follows: 0.91 (CI 0.88–0.93) for APACHE II, 0.87 (CI 0.83–0.90) for BISAP, 0.79 (CI 0.73–0.86) for CTSI, 0.80 (CI 0.72–0.89) for mCTSI, 0.87 (CI 0.81–0.92) for the Ranson score, and 0.73 (CI 0.66–0.81) for CRP. The APACHE II scoring system demonstrated a significantly higher predictive value for mortality compared to CTSI (*p* = 0.001) and CRP (*p* < 0.001). However, the predictive accuracy of CTSI was not significantly different from that of BISAP, mCTSI, CRP, or Ranson criteria.

For the prediction of AP severity, the AUC values were 0.79 (CI 0.72–0.86) for BISAP, 0.80 (CI 0.76–0.85) for CTSI, 0.81 (CI 0.75–0.87) for the Ranson score, 0.83 (CI 0.75–0.91) for mCTSI, and 0.73 (CI 0.64–0.83) for CRP, with all tools showing comparable performance [12].

Our findings indicate that CTSI and mCTSI scores offer excellent predictive accuracy for severe acute biliary pancreatitis, with AUC values of 0.93 and 0.94, respectively. By incorporating both clinical and imaging parameters, CTSI and mCTSI provide a comprehensive assessment, which likely accounts for their superior predictive performance across diverse patient populations, as demonstrated in our results.

Regarding the accuracy of predicting the severity of acute pancreatitis, CRP levels measured at 48 h emerged as a strong independent predictor of severe ABP, with an AUC of 0.92. This aligns with other published studies that identified CRP as a valuable biomarker for early risk stratification in acute pancreatitis [3].

Other markers, such as 48 h creatinine and the length of hospital stay, showed a good correlation with the severity of acute pancreatitis. However, this finding is somewhat predictable, which is why a more detailed analysis of these parameters was not performed.

Our analysis also showed that procalcitonin levels were significantly associated with ABP severity (r = 0.43, *p* < 0.0001). This observation is in line with meta-analyses recently published [13] indicating procalcitonin’s predictive value, high sensitivity, and diagnostic accuracy for severe acute pancreatitis. While its inclusion in our analysis aimed to emphasize its relevance in assessing disease severity, we acknowledge that its high prognostic value does not exclusively pertain to biliary AP. Additionally, discrepancies in the reported accuracy of ProCT, such as the lower accuracy mentioned in the study conducted by Kim et al. [10], likely arise from differences in study design, patient populations, and timing of measurement. These factors underscore the variability of ProCT’s diagnostic and prognostic utility, and we aimed to present both perspectives to provide a balanced view of its role in acute pancreatitis.

It was previously reported that procalcitonin, as evaluated at the time of hospitalization, is a more accurate indicator of SAP than CRP levels, APACHE II, and Ranson scores. SAP can be predicted with 86% accuracy with a procalcitonin strip test [14].

In univariate analysis, fasting blood glucose (mg/dL) (*p* = 0.0001) was an independent predictor for the severity of acute pancreatitis. A study published in 2018 [15] proved that hyperglycemia can cause inflammatory cytokines to be released, which can set off a chain of inflammatory reactions that can quickly result in multi-organ functioning impairment. In a different study involving clinical data from 3656 patients with AP, univariate and multivariate logistic regression analyses were conducted to investigate the association between blood glucose levels and the duration of hospital stay. Blood glucose levels and hospitalization no less than two days, no less than five days, and no less than seven days were found to be significantly nonlinearly correlated (all *p* < 0.001) [16]. Furthermore, Yan et al. [17] discovered a correlation between glucose levels and the death rate of AP patients admitted to hospitals.

In a study performed on 1046 AP cases split into a control group and a case group (SAP group), age, ICU admission, hypertension, hospital stay, CRP, leukocytes, BUN, and BISAP scores were evaluated in univariate analysis and were all significantly different between the case and control groups (*p* < 0.05) [18].

Similar to our results, in a study that evaluated the BISAP scoring system in prognostication of acute pancreatitis, the authors concluded that BISAP is highly effective in predicting severity, organ failure, and mortality in acute pancreatitis, outperforming the Ranson criteria, CTSI, and CRP levels [19].

Combined scoring models yield better predictive reliability than individual scores alone. They provide better clinical outcomes, increase predictive reliability, and enable more accurate patient classification. In multivariate analysis, we combined CTSI, BISAP, and CRP at 48 h into a multiparametric model, achieving a slightly higher AUC.

The combined diagnosis demonstrated higher specificity and sensitivity than either the independent use of BISAP, CRP, or NLR. The BISAP score, NLR, CRP, BISAP combined with NLR, and BISAP combined with CRP showed AUC values of 0.88, 0.81, 0.71, and 0.95, respectively, for predicting the severity of acute pancreatitis. The combined diagnostic approach demonstrated greater specificity and sensitivity compared to using the BISAP score, CRP, or NLR individually, similar to the findings of our study [20].

To further increase diagnostic and prognostic accuracy, future research should investigate further biomarkers and validate these findings across larger, more diverse cohorts.

Considering the valuable data this study offered, it should be noted that it has a number of drawbacks. It is a single-center, retrospective study that depends on an examination of current medical records. A bigger cohort would increase the study’s power and lower the possibility of mistakes, even though the sample size of 108 patients is adequate for preliminary analysis. The reliability of statistical relationships may be impacted by a small sample size, which also makes it more difficult to identify small variations across groups. Other factors, including medications (e.g., statins, anticoagulants) and lifestyle factors (e.g., alcohol use), could have affected the severity and outcomes of acute pancreatitis, even though a number of important variables were taken into consideration.

The multivariate analysis included established scoring systems such as BISAP and CTSI alongside clinical and laboratory parameters to evaluate their independent predictive value for disease severity. The goal was not to create a new composite score but to assess the relative contribution of each variable in predicting outcomes within our cohort. While this approach highlights the comparative utility of existing scores, we acknowledge its potential limitations in clinical practicality, as it involves the integration of multiple scoring systems. The analysis aimed to provide insights into the role of each variable rather than propose a directly implementable predictive model. Future research will focus on developing simplified models with strong predictive accuracy and greater applicability in routine clinical practice.

Another aspect that may be considered a limitation is related to the fact that the CTSI score demonstrated a high AUC (0.93) in our study. However, it is important to consider that the timing of the CT examination, typically performed later in the disease course compared to other scoring systems such as the BISAP score (assessed at admission) and the Ranson score (assessed at 48 h), plays a significant role in its performance. The delayed timing of CT imaging allows for a more accurate evaluation of local complications, such as pancreatic necrosis and peripancreatic fluid collections, which are pivotal in assessing severity. This protocol explains why, in our clinic, most patients undergo CT imaging at 48 h.

## 5. Conclusions

In conclusion, the markers proposed in this article play a pivotal role in assessing the severity of acute biliary pancreatitis. Individually, these markers provide valuable insights; however, their combined use in a multiparametric model enhances predictive accuracy. This integrative approach enables a more precise evaluation, aiding in the early identification of severe cases and facilitating timely, targeted interventions to improve patient outcomes.

## Figures and Tables

**Table 1:** Characteristics of the included subjects.

Parameter	Subjects n = 108
Mean age (years)	60.1 ± 18.6
Gender	
Males	37/108 (34.3%)
Females	71/108 (65.7%)
Mean BMI (kg/m^2^)	29.42 ± 5.23
AST (UI/L)	244 (21–1263)
ALT(UI/L)	256.5 (18–1157)
GGT (mg/dL)	319 (29–1143)
ALP (mg/dL)	153 (52–1222)
Total bilirubin (mg/dL)	2 (0.5–13.3)
FBG (md/dL)	97 (64–278)
CRP (mg/dL)	30.7 (4–424)
White blood cells (thousands/mm^3^)	11.7 (2.7–57.3)
CRP-48 (mg/dL)	81 (4.7–509)
Serum creatinine (mg/dL)	0.8 (0.3–8.5)
Serum creatinine (mg/dL) at 48 h	1.3 (0.3–8.7)
Serum urea (mg/dL)	38.5 (10–309)
BUN (mg/dL)	17.9 (4.7–97.5)
Procalcitonin distribution ranges (ng/mL)	
<0.5	137 (65.2%)
0.5–2	30 (14.3%)
2–10	13 (6.2%)
>10	30 (14.3%)
Comorbidities	
Complex cardiac diseases	25.9% (28)
HTA	37.9% (41)
CKD	4.6% (5)
Diabetes mellitus	29.6% (32)

Normal distributed numerical variables are reported as mean ± standard deviation, whereas those with a non-normal distribution are expressed as median and range. ALT—alanine aminotransferase, AST—aspartate aminotransferase, BMI—body mass index, GGT—gamma-glutamyl transferase, ALP—alkaline phosphatase, CRP—C-reactive protein, BUN—blood urea nitrogen, FBG—fasting blood glucose, CRP-48—CRP at 48 h, HTA—arterial hypertension; CKD—chronic kidney disease.

**Table 2:** Correlation between laboratory parameters, prediction scores, and the Atlanta classification of acute.

Parameter	Atlanta Classification of Acute Pancreatitis
Mean BMI (kg/m^2^)	r = 0.36, *p* < 0.0001, 95%CI (0.211–0.494)
AST (UI/L)	r = 0.21, *p* = 0.0249, 95%CI (0.116–0.364)
ALT(UI/L)	r = 0.23, *p* = 0.0216, 95%CI (0.118–0.381)
GGT (mg/dL)	r = −0.12, *p* = 0.8095, 95%CI (0.096–0.314)
ALP (mg/dL)	r = −0.17, *p* = 0.0720, 95%CI (−0.356–0.015)
Total bilirubin (mg/dL)	r = 0.29, *p* = 0.0022, 95%CI (0.108–0.455)
Fasting blood glucose (mg/dL)	r = 0.35, *p* = 0.0002, 95%CI (0.172–0.505)
CRP (mg/dL)	r = 0.36, *p* = 0.0002, 95%CI (0.175–0.509)
White blood cells (thousands/mm^3^)	r = 0.28, *p* = 0.0048, 95%CI (0.084–0.436)
CRP (mg/dL) at 48 h	r = 0.70, *p* < 0.0001, 95%CI (0.511–0.814)
Serum creatinine (mg/dL)	r = 0.38, *p* < 0.0001, 95%CI (0.235–0.534)
Serum creatinine (mg/dL) at 48 h	r = 0.49, *p* < 0.0001, 95%CI (0.371–0.623)
Serum urea (mg/dL)	r = 0.26, *p* = 0.0023, 95%CI (0.211–0.389)
BUN (mg/dL)	r = 0.29, *p* = 0.0017, 95%CI (0.196–0.413)
Procalcitonin levels	r = 0.43, *p* < 0.0001, 95%CI (0.331–0.529)
BISAP	r = 0.76, *p* < 0.0001, 95%CI (0.522–0.874)
CTSI score	r = 0.66, *p* < 0.0001, 95%CI (0.480–0.775)
mCTSI score	r = 0.68, *p* < 0.0001, 95%CI (0.510–0.853)
Ranson score	r = 0.61, *p* < 0.0001, 95%CI (0.458–0.734)
Duration of hospitalization (days)	r = 0.43, *p* < 0.0001, 95%CI (0.266–0.576)

BISAP—Bedside Index of Severity of Acute Pancreatitis, CTSI—CT severity index, mCTSI—modified CT severity index.

**Table 3:** Multivariate regression analysis for predicting the severity of acute pancreatitis.

Predictors	Regression Parameters		
	β	SE	*p*
CTSI	β = 0.3265	±0.00281	*p* < 0.0001
BISAP	β = 0.1298	±0.00039	*p* = 0.0082
CRP at 48 h	β = 0.1148	±0.00131	*p* = 0.0091

**Table 4:** Predictive accuracy of the multiparametric model compared with that ofeach individual score, as well as among individual scores.

**MM vs. mCTSI**	
Differencebetweenareas	0.0324
zstatistic	1.058
Significancelevel	*p* = 0.2901
**MM vs. BISAP**	
Differencebetweenareas	0.0580
zstatistic	2.149
Significancelevel	*p* = 0.1316
**MM vs. CRP**	
Differencebetweenareas	0.0455
zstatistic	1.419
Significancelevel	*p* = 0.1560
**MM vs. CTSI**	
Differencebetweenareas	0.0335
zstatistic	1.119
Significancelevel	*p* = 0.2632
**mCTSI vs. BISAP**	
Differencebetweenareas	0.0256
zstatistic	0.866
Significancelevel	*p* = 0.3863
**mCTSI vs. CRP-48**	
Differencebetweenareas	0.0131
zstatistic	0.535
Significancelevel	*p* = 0.5928
**mCTSI vs. CTSI**	
Differencebetweenareas	0.00114
zstatistic	0.0609
Significancelevel	*p* = 0.9515
**CRP-48 vs. BISAP**	
Differencebetweenareas	0.0125
zstatistic	0.417
Significancelevel	*p* = 0.6766
**BISAP vs. CTSI**	
Differencebetweenareas	0.0244
zstatistic	0.952
Significancelevel	*p* = 0.3413
**CRP-48 vs. CTSI**	
Differencebetweenareas	0.0119
zstatistic	0.837
Significancelevel	*p* = 0.4027

**Table 5:** Classification of subjects according to the MM and the proposed scores.

**Correctly Classified Subjects**		**Classification Model**			
		**I. BISAP**	**II. CRP-48**	**III. CTSI**	**IV. MM**
		75% (30/40)	72.5% (29/40)	75% (30/40)	95% (38/40)
***p* Values**					
I/II	I/III	I/IV	II/III	II/IV	III/IV
1	0.796	0.028	0.162	1	0.028

## Data Availability

Data are available upon request by contacting the corresponding author at the following email address: bende.renata@umft.ro.
